# Semen Quality and Sperm Function Loss by Hypercholesterolemic Diet Was Recovered by Addition of Olive Oil to Diet in Rabbit

**DOI:** 10.1371/journal.pone.0052386

**Published:** 2013-01-11

**Authors:** Tania E. Saez Lancellotti, Paola V. Boarelli, Aida A. Romero, Abi K. Funes, Macarena Cid-Barria, María E. Cabrillana, María A. Monclus, Layla Simón, Amanda E. Vicenti, Miguel W. Fornés

**Affiliations:** 1 Laboratorio de Investigaciones Andrológicas de Mendoza (LIAM), Instituto de Histología y Embriología (IHEM), Facultad de Ciencias Médicas, Universidad Nacional de Cuyo, Centro Científico Tecnológico (CCT) – Consejo Nacional de Investigaciones Científicas y Técnicas (CONICET), Mendoza, Argentina; 2 Instituto de Investigaciones, Facultad de Ciencias Médicas, Universidad del Aconcagua, Mendoza, Argentina; National Cancer Institute, United States of America

## Abstract

Fat increment (0.05% cholesterol, chol) in standard diet promoted a significant increase in serum and sperm membrane chol, which ultimately altered membrane-coupled sperm specific functions: osmotic resistance, acrosomal reaction, and sperm capacitation in White New Zealand rabbits. These changes were also associated with a reduction in motility percentage and appearance of abnormal sperm morphology. The present study was carried out to evaluate the effect of dietary olive oil (OO, 7% v/w) administration to several male hypercholesterolemic rabbits (hypercholesterolemic rabbits, HCR) with altered fertility parameters. These HCR males were achieved by feeding normal rabbits with a high-fat diet (0.05% chol). HCR were associated with a modest non-significant increase in body weight (standard diet, 4.08±0.17 Kg, versus high-fat diet, 4.37±0.24 Kg). Hypercholesterolemic rabbits presented a marked decrease in semen volume, sperm cell count, and percentage of sperm motility, associated with a significant increase in sperm cell abnormalities. Moreover, sperm capacitation measured by the characteristic phosphorylated protein pattern in and induced acrosomal reaction were also altered suggesting sperm dysfunction. However, the administration of OO (for 16 weeks) to rabbits that were fed with 50% of the high-fat diet normalized serum chol. Curiously, OO supply succeeded to attenuate the seminal and sperm alterations observed in HCR group. Administration of OO alone did not cause any significant changes in above mentioned parameters. These data suggest that OO administration to HCR male rabbits recovers the loss of semen quality and sperm functionality.

## Introduction

The relationship between obesity/hypercholesterolemia and reduced male fertility has been reported clinically and in experimental models [1]–[12]. The effect of high cholesterol (chol) intake and its impact in different tissue/organs has also been described in several models [13]. The deleterious impact on reproductive tissues has been studied in rabbits and other species [6], [7], [10], [14]–[18]. Changes in lipid content in testis [19] and epididymal cells [20] were reported in normal or genetically reprogrammed animals. But few papers were focused on the addition of natural products in the food as a protective diet, in order to avoid sperm alteration [14], [15].

In the experimental setting, male animals fed with a high-fat diet were associated with deleterious changes in semen and sperm cells. Alterations reported include decreased volume semen and sperm number associated with an increase in sperm abnormal morphology in rabbit and mouse models [12], [16], [21], [22]. Furthermore, fat-enriched diets - fat from animal source - also had an impact on cell function, such as sperm cell functionality [12], [22]. Changes in sperm membrane chol concentration and distribution lead to alteration of membrane-coupled sperm specific functions: sperm motility, membrane osmotic resistance to hipoosmotic shock, sperm capacitation and induced acrosomal reaction (AR) were all significantly reduced; probably due to an increase in membrane chol content [12]. Capacitation and AR are processes required for fertilizing the oocyte *in vivo*. During sperm capacitation, a number of changes occur at the sperm surface such as membrane protein and lipid re-organization. These changes are likely to result in the capacitated state which characteristically allows the sperm to bind the pellucid zone and immediately thereafter to acrosome react [23]–[25].

Olive oil, a component of Mediterranean diet, has been proposed by several studies as a protective agent against vascular injury promoted by acquired hypercholesterolemia [26]. Interheart studies [13], population studies [27] and animal models [28], [29] analyzed the negative effect of chol serum increase and the corresponding protection by OO administration.

In the present report we took advantage of our established rabbit model of diet-induced hypercholesterolemia [12] to assess whether OO administration can recover semen and sperm parameters altered in dietary acquired hypercholesterolemia. We found that supplementing fat diet with OO improved not only serum cholesterol level but also semen quality and sperm function.

## Materials and Methods

### Ethics statement

The animal studies described here were reviewed and approved by the animal care and use committee of School of Medicine, National University of Cuyo (Institutional Committee for Use of Laboratory Animals, IACUC [30]).

### Reagents

Unless otherwise stated, all chemicals and solvents of the highest grade available were obtained from Sigma (St. Louis, MO, USA) and Merck (Darmstadt, Germany). Olive oil (OO) used corresponds to virgin OO.

### Animals and diets

**Figure 1:**
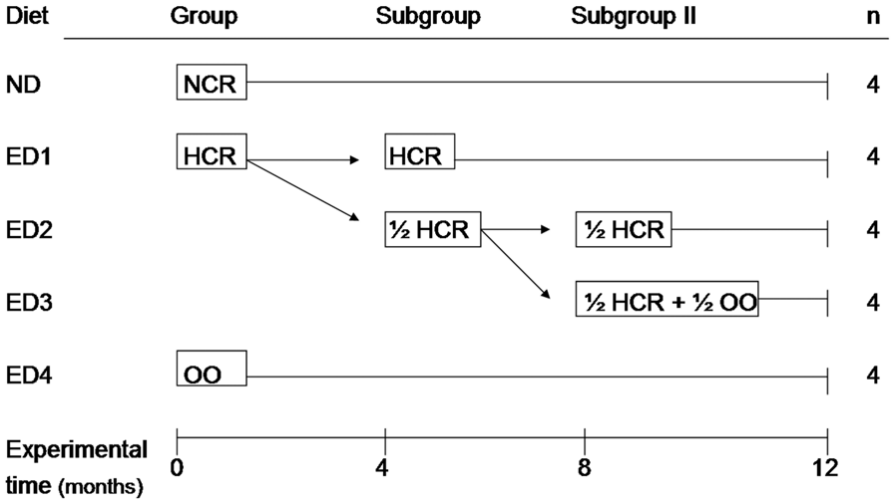
Experimental groups. Twenty New Zealand rabbits were initially distributed at random in three groups: NCR, HCR, OO at 5 months of age (corresponding to experimental time (et) = 0 months). After 4 months, NCR continued with normal diet (ND) and HCR was split in two subgroups: HCR and ½ HCR. HCR continued with experimental diet 1 (ED1) and the last group was fed with ED2. Four months later (et = 8 m) ½ HCR was divided again in two (subgroup II): ½ HCR (fed with ED2) and ½ HCR+½ OO (fed with ED3). m = months, n = number of experimental animals. More details in the text.

For the purposes of this study, twenty fertile male White New Zealand rabbits (5–17 months old of age, acquired from “Don Cipriano” farm, Mendoza, Argentina) were caged individually during 12 months with a photoperiod of 12 hours light/day and a temperature ranging from 18–25°C. Animals were fed *ad libitum* with a standard rabbit diet following our previous animal model [12]. At five months of age (experimental time = 0 months), rabbits were divided into two groups (4/12 animals each) maintaining the average of body weight in both experimental groups. The first group (4 animals), which served as control (designated normal cholesterolemic rabbits, NCR, Figure 1), continued fed with standard cereal-based chow for this specie during the entire experiment (normal diet, ND; composed of 17% crude protein, 60.5% carbohydrates, 16% fiber, 0% saturated fat, 5.3% minerals, and 12% water; data from the manufacturer's analysis, GEPSA FEEDS®). The other group (12 animals, hypercholesterolemic rabbits, HCR, Figure 1) was fed with experimental diet 1 (ED1), consisting in 15% crude protein, 14% fiber, 13.5% fat (6.5% saturated fat, 0.05% cholesterol). After 4 months, HCR group was split in two (4/8 animals each). The first subgroup, HCR, continued with ED1. The rest of animals were fed with experimental diet 2 (ED2) that corresponds to 50% fat of ED1 (½ HCR subgroup, Figure 1). The fat reduction to 50% in this diet allows subsequent olive oil incorporation to the chow in the next step to conform the subgroup II named ½ HCR+½ OO (see below). All animals were checked for serum chol concentration weekly. When animal consuming ED2 decreased significantly their serum chol level compared to HCR, they were split again in two second subgroups (subgroup II, 4 animals each); one of them continued with the fat reduction (½ HCR, ED2) and the other was fed with the same fat-reduced diet (ED2) plus 7% OO v/w (ED 3, Figure 1). The last group was named ½ HCR+½ OO. Finally, other group was set up consisting in four rabbits maintained with a standard diet supplemented with OO to study OO impact. Concentration of olive oil used in OO diet corresponds to twice olive oil volume (14% v/w, ED4) trying to reach the same total lipid content of ED1.

During the study period of 12 months, rabbits received daily experimental diets and water ad libitum. Food intake and body weight were recorded weekly (data not shown).

### Plasma lipids

Plasma chol was determined from their arrival to our animal facility. Blood samples were collected from the marginal ear vein of non-anaesthetized animals, with heparinized syringes. Plasma was isolated after centrifugation at 800 *g* for 10 min. Plasma chol concentration was determined using GT Lab kit under manufacturer's instructions (CHOD/PAP, GT Lab). The initial level of plasma chol and body weight showed no significant differences between animals.

### Cholesterol analysis

Total lipids from recently ejaculated sperm (from all rabbit groups/subgroups) after washing with phosphate buffer saline (PBS) were extracted following the instructions indicated by L.S.E.(Laboratorio de Servicios y Ensayos, I.N.T.I. – Frutas y Hortalizas, Luján de Cuyo, Mendoza, Argentina). Phosphate buffer saline was obtained diluting a Sigma tablet in 200 ml pure water. Final concentration: 0.01 M phosphate buffer, 0.027 M KCl, 0.137 M NaCl, pH 7.4). Cholesterol concentration was determined by Gas Chromatography (AutoSystem XL, Perkin Elmer) and is reported with respect to 1×10^9^ spermatozoa.

### Membrane cholesterol detection

Non-capacitated sperm cells were fixed with 4% paraformaldehyde in PBS 30 min at room temperature. Samples were then washed three times with PBS and centrifuged at 800 *g* 10 min. Sperm pellets were incubated with 0.15 mM of filipin complex 60 min in PBS protected from light [12]. Cells were mounted with PBS-glycerol (50% v/v) for fluorescence microscopy analysis (380 nm Ex and 475 nm Em, Nikon Optiphot). Sperm fluorescence was observed and recorded with a Hamamatsu C4742-95 camera connected to an inverted microscope NIKON TE-2000. Sperm head fluorescence intensity was estimated by Image J software [31].

### Semen collection and handling

Ejaculated semen was collected by an artificial vagina (with a device built by us following commercial models, [32], [33]) from fertile New Zealand rabbits (6–17 month old), in accordance with the Guide for Care and Use of Laboratory Animals [34]. Two ejaculates were monthly obtained from each male, and then stored at 37°C until evaluation 15 minutes after collection.

### Semen samples

Sperm were evaluated and capacitated under conditions explained in Saez et al, 2010 [12]. The results shown in all the figures and table were performed during the last 4 months of experimental time, unless otherwise stated.

### Semen evaluation

Samples containing urine and cell debris were discarded whereas gel plugs were removed. Semen samples were immediately assessed for physical parameters as aspect, color, volume and pH. Percentages of viability and morphological abnormalities were determined after a vital eosin stain [35], eosin 1% was prepared by diluting Y eosin in PBS. Non stained cells were considered alive and expressed as percentage of total sperm cell counted in 40 µl of semen. Cell counting was performed on a slide mixing equal drops of semen and eosin solution (final concentration 0.5%) under 400× magnification in a bright field microscope. This microscopy preparation was also used to evaluate sperm morphology [36]. In all cases 200 sperm cells were counted. After that, semen samples from all groups were diluted (1∶50, v/v) with warmed PBS and sperm motility of diluted samples was evaluated at 250× under a phase-contrast microscope maintained at 37°C. Motility (progressive and *in situ*) was expressed as percentage of motile sperm over 200 cells. At the same time, cell concentration of diluted samples was estimated using a Macler counting chamber (Sefi-Medical Instruments, Israel). Finally, semen remaining samples were washed twice by centrifugation - resuspension at 600 *g* for 10 min in PBS to remove seminal plasma. The final pellet was resuspended with PBS (20 to 200 µl, depending on the pellet volume).

### Sperm capacitation

Briefly, sperm suspension was adjusted to 5–10×10^6^ cells/ml with BWW medium [37] and split for incubation in two 35 mm Petri dishes (Corning®) under conditions that support or not capacitation during 16 h. One dish was supplemented with 4 mg/ml Bovine Serum Albumin, BSA, fraction V, corresponding to BSA +, capacitated conditions. The other dish was incubated with BWW alone, BSA −, non-capacitated conditions. All dishes also contained 20 mM NaHCO_3_, and the atmosphere corresponds to 37°C, 5% CO_2_, 95% air. Sperm capacitation was determined by phospho-tyrosine (p-Y) proteins [38], [39], see below in phospho-tyrosine evaluation.

### Membrane integrity

Plasma membrane integrity was evaluated by Hypo-Osmotic Test (HOST-T, [40]. Sperm cells were incubated in a hypo-osmotic solution (25 mM sodium citrate, 75 mM fructose in double distilled water) for 30 min at 37°C and then evaluated under phase contrast microscopy described in Saez et al., 2010 [12]. Swelling of sperm cells was identified as changes in the shape of the tail. At least 100 cells were counted, and the percentage of spermatozoa that showed swelling in the hypo-osmotic solution corresponds to not damaged spermatozoa (percentage of swollen spermatozoa) indicating membrane integrity.

### Phospho-tyrosine evaluation (sperm capacitation status)

Following an incubation period of 16 h, sperm cells were concentrated by centrifugation 15 min 850 *g* at room temperature, washed twice in 1 ml PBS containing 0.2 mM Na_3_VO_4_, and then resuspended in sample buffer [41] without mercaptoethanol boiling for 5 min. After centrifuging at 10,000 *g* for 15 min, the supernatant was removed and frozen until used. Five percent of 2-mercaptoethanol was added to defrosted samples and they were subjected to SDS-PAGE using 8–10% mini-gels according to Laemmli [41]. Protein extracts loaded per lane were equivalent to 5–10×106 sperm. Each gel contained dual-prestained molecular weight standard (Biorad, Hercules, CA). Proteins were transferred to 0.45 µM nitrocellulose membranes (Bio Rad) and nonspecific reactivity was blocked by incubation over night with 3% Teleostean fish gelatin dissolved in washing buffer (TTBS, Towbin's buffer plus 0.1% Tween 20). Blots were incubated with the anti-Phopho-Tyrosine antibody 1∶5000 (ICN Biomedicals) in blocking buffer for 1 h, RT. Biotin-conjugated anti-mouse IgG (Sigma) was used as secondary antibody (1∶1250) and Horseradish peroxidase-conjugated extravidine (Sigma) was added at the end (1∶750), both with a period of incubation of 1 h, at room temperature. Excess first and second antibodies were removed by washing three times for 10 min each in TTBS. Detection was accomplished with an enhanced chemiluminescence system (ECL; Amersham Biosciences) and subsequent exposure to Blue Sensitive Cole-Parmer X-ray films (Cole-Parmer Instruments Company) for 5–30 s.

### Acrosome reaction (AR) assay

Capacitated sperm were incubated 15 min 37°C with (induced reaction) or without (spontaneous reaction) progesterone (10 µM progesterone in DMSO), [42]. AR was evaluated by Triple Stain Technique [12], [43]. At least 300 cells were scored from each rabbit (in all conditions) to evaluate acrosomal reaction. For each experiment, AR percentage was calculated as percentage of reacted sperm over 300 sperm cells as: (Number of reacted sperm induced by progesterone - Number of spontaneously reacted sperm)×100/300 = AR percentage. This percentage was first established for NCR group and considered the control AR status. AR index was calculated as a percentage of this value (AR index). In this way AR index expresses the 100% for sperm from NCR.

### Statistical analysis

Data were analyzed using statistical packages software GraphPad Prism 4 [44]. Unless otherwise expressly noted, results in the text, tables, and graphs are reported as means ± SEM of at least three independent experiments performed in duplicate. Differences between groups were evaluated by the Student's t-test considering a p value of less than 0.05 as statistically significant.

## Results

### Effect of dietary treatments on body weight

In the present study, rabbits under experimental diets (HCR, OO, ½ HCR, ½ HCR+½ OO) were not significantly fatter than NCR during the experiment. The mean weight at the end of the study (standard diet, 4.08±0.17 Kg, versus high-fat diet, 4.37±0.24 Kg) was used to estimate body mass index (BMI), which did not present difference between groups, resulting in non-obese animals (data not shown).

### Effect of diet on blood cholesterol

**Figure 2:**
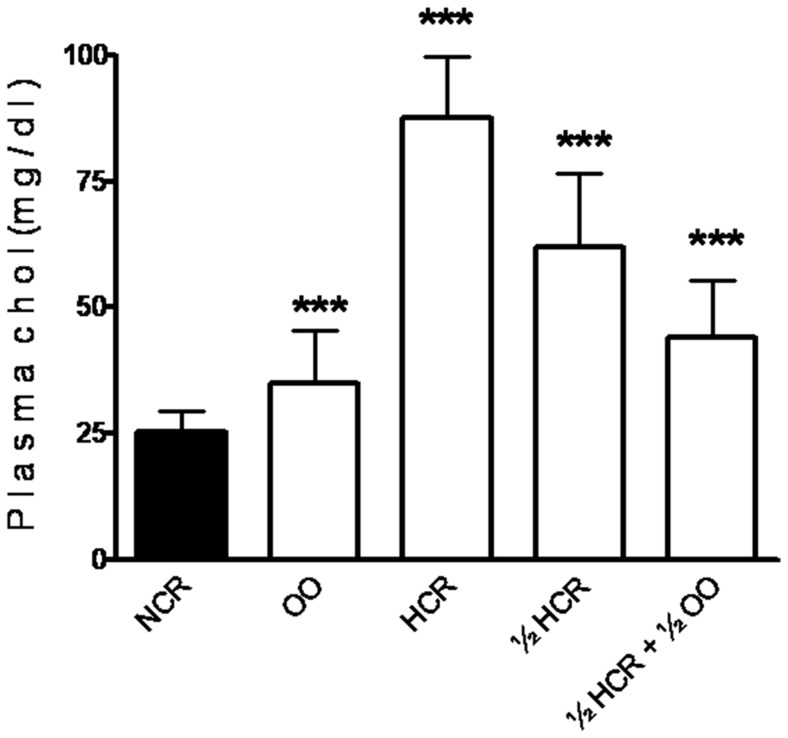
Effect of diet on blood cholesterol. Bars represent the mean ± SEM of plasma cholesterol concentration from NCR, OO, HCR, ½ HCR and ½ HCR+½ OO during the last 3 months of experiment. n = 45 samples. *** indicates significantly different from NCR (p<0.001).

Male rabbits provided with a high-fat diet (ED1, HCR) were associated with a significant elevation of blood cholesterol (25.22±4 mg/dl for NCR, 87.53±12 mg/dl for HCR; Figure 2). When the fat intake was reduced to 50% (ED2, ½ HCR), the serum chol decreased (62±14 mg/dl). But the addition of 7% OO (v/w) to ED2 (ED3, ½ HCR+½ OO) leads to a decrease in serum chol level (62±14 mg/dl) reaching a concentration that approximates OO group (35±10 mg/dl), but did not reached normal level seen in control rabbits (ND, NCR). These data demonstrate that male rabbits fed a high-fat diet become hypercholesterolemic without the development of overt obesity. OO therapy in male rabbits fed a high-fat diet has a beneficial effect on the progression of hypercholesterolemia.

### Effect of dietary treatment on semen parameters

We have previously seen that feeding cholesterol had a deleterious effect over semen parameters and sperm functionality [12]. Data concerning semen volume, pH, sperm number, viability, motility and morphology are presented in table 1. The difference in semen volume, pH and sperm viability between all groups/subgroups was not significant (Table 1). Sperm concentration and percentage of motile spermatozoa were significantly decreased in HCR group (ED1) and ½ HCR subgroup (ED2) than NCR group (Table 1), and also when compared to ½ HCR+½ OO (data not shown, ED3). Differences in the parameters mentioned above between HCR group and ½ HCR subgroup were not significant (data not shown). We found that semen parameters in ejaculated samples in rabbits fed with OO (OO group, ED4), were not significantly different from NCR. It was also found that after administration of olive oil, semen parameters affected by hypercholesterolemia (½ HCR) significantly improved.

**Table 1:** General characteristics of fresh rabbit semen samples (mean ± SEM).

	GROUPS			SUBGROUP	SUBGROUP II
	NCR	OO	HCR	½ HCR	½ HCR+½ OO
Volume (µl)	759.8±68.66	754.5±42.37	432.2±45.6*	471.2±33.42*	643±45.6
pH (media ± SD)	7.5±0.5	7.5±0.5	7.5±0.25	7.5±0.5	7.5±0.5
Sperm viability after eosin staining (%)	88.8 7±1.28	88.2±1.5	85.8±1.15	87±1.6	89±1.3
Sperm concentration (10^6^/ml)	629.2±90.62	703.1±59.5	521±118.4	510±48.1	730±138
Total sperm motility (% A+B+C)	76.8±3.3	78.3±7	54.6±4.1*	55±5*	71±6
Total sperm abnormalities (%)	21.1±2.4	20.2±3	33.6±3.5*	32.8±5*	23±5

* p<0.05, n = 25.

### Effect of dietary treatment on sperm membrane cholesterol

**Figure 3:**
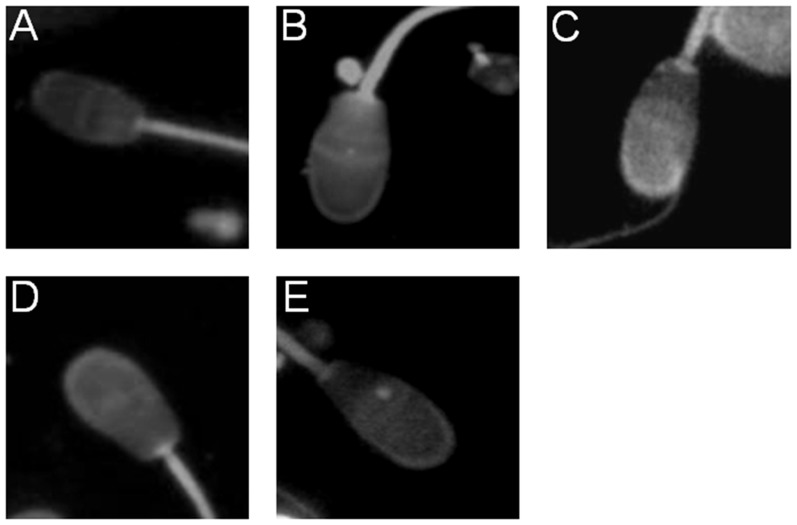
Effect of dietary treatment on sperm membrane cholesterol. Fluorescence micrographs showing cholesterol content in plasma membrane of ejaculated rabbit spermatozoa detected by filipin probe. Images correspond to filipin-stained sperm cells (×630) from NCR (A), OO (B) HCR (C), ½ HCR (D) and ½ HCR+½ OO (E). Compare the strong signal detected in HCR (C) with the one from the ½ HCR+½ OO (E). The experiment was performed at least three times with 20 sperm from each animal.

Sperm membrane cholesterol was assessed qualy- and quantitatively by filipin fluorescence (Figure 3) and gas chromatography respectively. Notably, sperm from ½ HCR+½ OO rabbits show less fluorescence intensity (Figure 3 E) compared to ½ HCR (Figure 3 D) and HCR (Figure 3 C), and moderately increased when compared to NCR (Figure 3 A) sperm cells. OO addition to high fat diet reduced the total cholesterol content of spermatozoa at the end of the study: 73 µg/10^9^ cells for ½ HCR+½ OO, 110 µg/10^9^ cells for HCR and 64 µg/10^9^ cells for NCR.

### Impact of diet on sperm functionality

**Figure 4:**
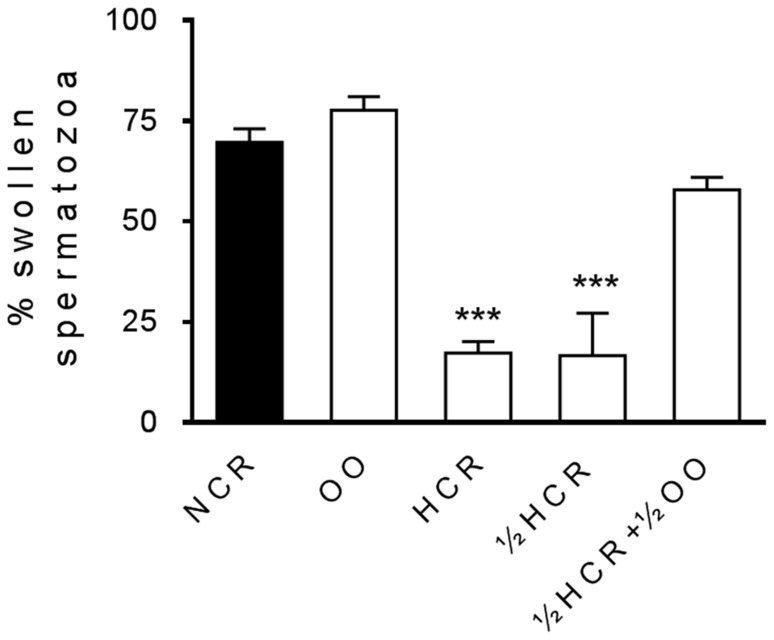
Impact of diet on sperm functionality: membrane response to hypo-osmotic swelling test. Bars represent the percentage (mean ± SEM) of spermatozoa swollen from NCR (black bar), OO, HCR, ½ HCR and ½ HCR+½ OO (white bars). The experiment was performed at least three times with each animal. n = 25 samples. *** = significantly different from NCR (p<0.001).

**Figure 5:**
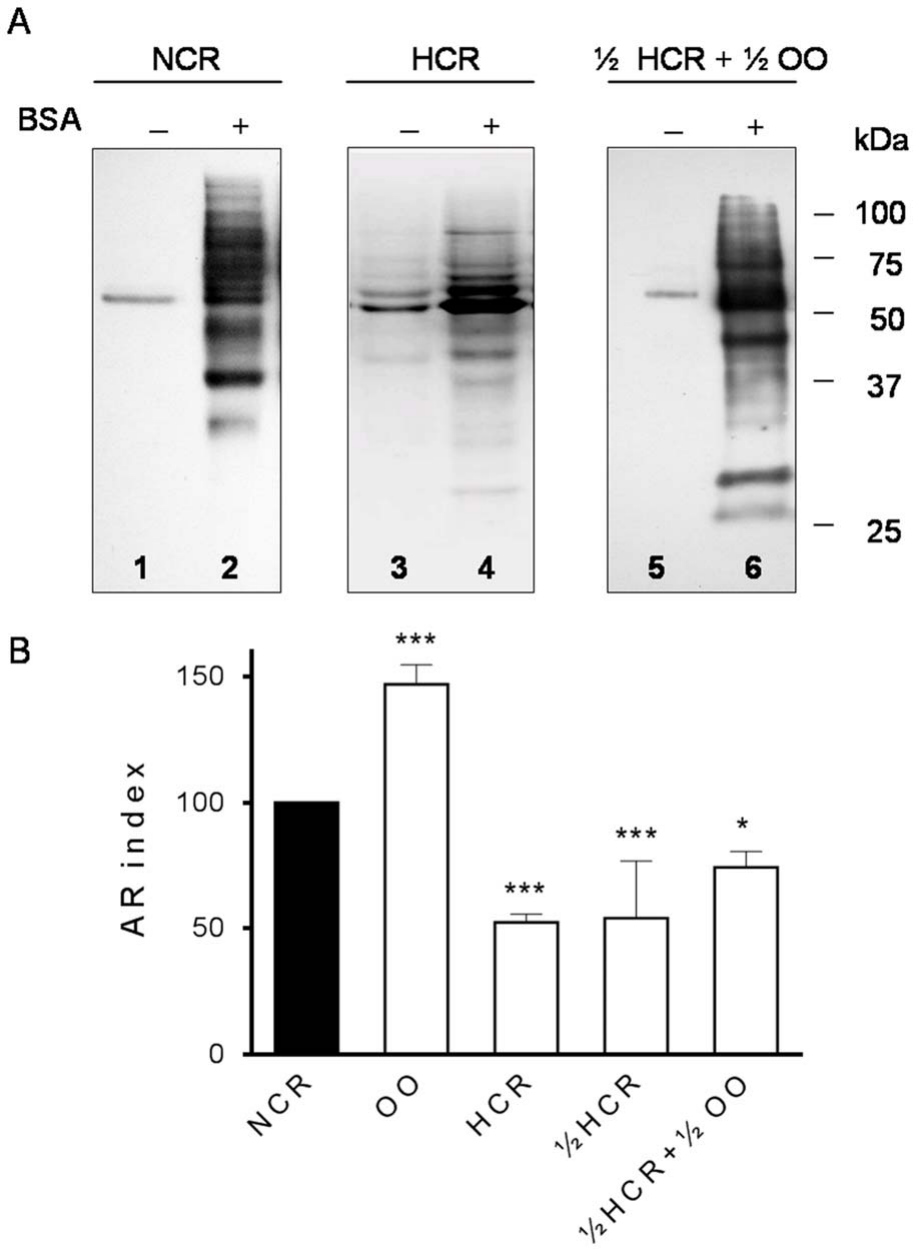
Impact of diet on sperm functionality: sperm capacitation and acrosome reaction. Protein tyrosine phosphorylation (A) and acrosomal exocytosis index (B) of rabbit spermatozoa. A: phospho-Y proteins from control (NCR), HCR and ½ HCR+½ OO showed different patterns ranging from one band (non-capacitated, culture medium without BSA (−), approximately 60 kDa) to many bands (capacitated with BSA (+), from over 20 to 100 kDa). Notice that capacitated sperms from HCR decreased the p-Y protein pattern compared with NCR, and ½ HCR+½ OO shows a p-Y pattern resembling control (NCR) conditions. The experiment was performed at least three times and representative blot is shown. B: Bars represent AR index of spermatozoa from NCR, ½ HCR, ½ HCR+½ OO and OO after 10 µM progesterone incubation. AR index corresponds to normalized data (see Materials and Methods). n = 25 samples. *** = significantly different from NCR (p<0.001).

Fat diet (ED1) induced dramatic deleterious effects on functional parameters of sperm cells [12], also seen with the reduction of 50% fat (ED2) in ½ HCR group (figures 4 and 5 B). Curiously, when maintaining fat reduction with the concomitant adition of 7% OO (½ HCR+½ OO) for 4 months, spermatozoa showed a significant recovery in sperm parameters affected by fat consumption: membrane response to the hypo-osmotic swelling test significantly improved (Figure 4) compared to the membrane impairment we had previously described for HCR and also evidenciated in this work for ½ HCR. Sperm from ½ HCR+½ OO also enhanced protein tyrosine phosphorylation (p-Y) signal under capacitation conditions (Figure 5 A) and achieved a pattern of p-Y protein bands similar to control (NCR). Concerning acrosome reaction, sperm from ½ HCR showed impaired rate similar to HCR group when compared to NCR (Figure 5 B). On the other hand, the decrease in cholesterol concentration in plasma membrane of spermatozoa from ½ HCR+½ OO group (Figure 4 E) was consistent with an improvement in the AR rate (Figure 5 B). Curiously, sperm from OO group showed AR index significantly increased compared to NCR.

## Discussion

In hypercholeterolemic rabbit there was not only a marked decrease in semen volume and sperm cell count, but also a decline in the percentage of sperm motility associated with a significant increase in sperm cell abnormalities. Moreover membrane-coupled sperm specific functions: osmotic resistance, acrosomal reaction and sperm capacitation were also altered as it was reported previously [12], [21]. But, for the first time, this study demonstrated that dietary olive oil added to fat diet recovers the loss of semen quality and sperm function.

Effects of hypercholesterolaemia on Leydig and Sertoli cell secretory function and the overall sperm fertilizing capacity in the rabbit [45] and other species [46] have been reported previously. Moreover, an increase in the chol level in sperm membrane could cause a variation in the membrane rigidity that could modify the sperm membrane fusion capacity and functionality, as the diminished capacity of rabbit sperm to undergo the AR [14]–[16]. But in human sperm there are reports indicating that hypercholesterolaemia has no effect on cholesterol and phospholipid levels in spermatozoa and does not cause serious modification of the secretory function of the accessory sex glands [47]. In contrast, other authors agree with our results, claiming that cholesterol intake promotes several changes in the sperm cell [48].

The administration of OO (for 16 weeks) to rabbits that were fed with 50% of the high-fat diet, that already present a reduction in serum cholesterol, continued with the marked decrease in serum cholesterol. Moreover, OO supply succeeds to attenuate the seminal and sperm alterations observed in HCR and ½ HCR. The relationship between hypercholesterolemia and cardiovascular disease risk has been well established clinically in mildly hypercholesterolemic subjects, similar to the conditions studied here [49] and in experimental models [50] but attention has not been directed to fertility. Results presented here support the general agreement of benefice's of OO like as component Mediterranean diet [51].

The significant reduction in sperm concentration, percentage of motile spermatozoa, motility and the increment in the number of sperm abnormalities in ejaculated semen samples in animals with hypercholesterolaemia (HCR/½ HCR) had been previously reported [12], [21]. Here we emphasize that sperm alterations depend on the level of fat intake, less grease correspond to less affectation. Moreover, all semen parameters affected in hypercholesterolemic rabbits improved significantly when animals were also fed with OO (½ HCR+½ OO). The last subgroup did not present significant differences in the sperm or seminal parameters studied here with NCR. The present study also suggests that spermatozoa from hypercholesterolemic rabbits (HCR/½ HCR) have lower fertilizing capacity, as we show that hypercholesterolemia have a detrimental effect on the functions required to efectively fertilize the oocite, sperm capacitation and AR. This is consistent with previous reports [12], [21], suggesting that alterations in the sperm membrane lipids probably interfere with the intracellular cascades.

Morphological alteration of sperm cell could be attributable to abnormal spermatogenesis under fat increment. It was reported that lipid increment in testis from obese mouse promotes apoptosis and increment in lipid droplets in spermatogonial cells [20]. But there are no studies to describe ultrastructural sperm abnormalities that are generated as a result of increased fat supply in this particular environment. Moreover, the present study is the first to suggest a beneficial effect of olive oil on abnormal sperm. Further studies are necessary to discover the mechanism that is responsible for the latter effect. Sperm motility is a result of complex force acting at the sperm flagellum. Ultrastructural minimal modifications or futile metabolism changes could be the trigger in this rabbit model that explains the reduction of sperm motile percentage. Other papers also found the loss of motility under fat/cholesterol increment [21] but, again, the introduction of olive oil needs a great effort to understand the underline mechanisms.

Here it was demonstrated for the first time that OO administration promotes changes in sperm membrane chol concentration demonstrated here by two independent methods. These changes ultimately alter membrane-coupled sperm specific functions: sperm motility, membrane osmotic resistance to hipoosmotic shock, sperm capacitation and induced acrosomal reaction.

Cholesterol could be related with the rigidity of cell membranes [52]. In sperm cells cholesterol is highly expressed and compartmentalized [53], and when it is experimentally over expressed (as in our model), interferes with the hiposmotic test designed to check the functionality of the membrane [40]. Curiously the hypercholesterolemia under fat intake increment promotes sperm cell damage but not the olive oil intake alone. On the contrary fat and olive oil intake simultaneously decrease the cholesterol detection at the membrane (see filipin or chromatography results) and recover the host test. These results clearly indicate a recovery of normal sperm physiology.

We should emphasize here that cholesterol is crucial for rabbit sperm capacitation [54] and during epididymal preparation of spermatozoa that will then be ready to capacitate [55]. Capacitation was regulated by a signal transduction pathway beginning with loss of cholesterol from the plasma membrane [56]. The biological marker of this sperm capacitated status was the pattern of protein tyrosine phosphorylated [57]. Any increase in membrane cholesterol could interfere with the cascade as it was proposed early [12]. In these sense, similar results to previous report were obtained under hypercholesterolemia (HCR as well as ½ HCR groups) conditions. But capacitation of sperm from olive oil-protected rabbit show a p-Y pattern that corresponds to capacitation, but a different set of proteins appear phosphorylated. A clear explanation of this interesting phenomenon could not be defined yet.

AR is a processes required for sperm to fertilize the oocyte in vivo. A number of changes occur at the sperm surface during sperm capacitation such as membrane protein and lipid re-organization. It is these changes that are likely to result in the capacitated state which characteristically allows the sperm to bind to the pellucid zone and immediately thereafter to acrosome react [23]–[25], [58]. Supplementation of the diet with olive oil promotes a recovery of acrosomel index values close to the normal diet.

Recently, treatment of infertility by natural products has been topic of interest [59]–[62]. Olive oil, the principal fat of Mediterranean diet, is known to improve several cardiovascular risk factors at relatively high doses together with intensive modifications of dietary habits [49], [63]. Olive oil contains 77% monounsaturated fatty acids, 14% saturated fatty acids, and 9% polyunsaturated fatty acids, plus vegetable mucilage and Vitamin E. Monounsaturated fatty acids are far less easily damaged by oxygen than other types of fat. They are therefore less likely to produce free radicals, which damage many biological molecules: proteins, DNA and membrane lipid peroxidation [64]. Administration of OO may improve sperm quality probably by modifying the sperm lipid composition: The reduction in membrane cholesterol content certainly affected the phospholipids/cholesterol ratio of the sperm bilayer.

These data together demonstrate that daily OO supplementation together with a fat diet to male rabbits can restore the hypercholesterolemic response to basal level with a significant improvement of semen quality.
